# Development of 14 Microsatellite Markers for Zoonotic Tapeworm *Dibothriocephalus dendriticus* (Cestoda: Diphyllobothriidea)

**DOI:** 10.3390/genes11070782

**Published:** 2020-07-12

**Authors:** Eva Bazsalovicsová, Gabriel Minárik, Katarína Šoltys, Alžbeta Radačovská, Jesper A. Kuhn, Egil Karlsbakk, Karl Skírnisson, Ivica Králová-Hromadová

**Affiliations:** 1 Institute of Parasitology, Slovak Academy of Sciences, Hlinkova 3, 04001 Košice, Slovakia; bazsal@saske.sk (E.B.); kolenicova@saske.sk (A.R.); 2 Medirex, a.s., Galvaniho 17/C, P.O. Box 143, 82016 Bratislava, Slovakia; gabriel.minarik@gmail.com; 3 Faculty of Natural Sciences, Comenius University in Bratislava, Ilkovičova 6, 84215 Bratislava, Slovakia; katarina.soltys@gmail.com; 4 Freshwater Ecology Group, Department of Arctic and Marine Biology, Faculty of Biosciences, Fisheries and Economics, UiT The Arctic University of Norway, N-9037 Tromsø, Norway; jesperkuhn@gmail.com; 5 Department of Biological Sciences, University of Bergen, P.O. Box 7803, N-5020 Bergen, Norway; Egil.Karlsbakk@uib.no; 6 Institute for Experimental Pathology, University of Iceland, Keldnavegur 3-112, IS-112 Reykjavík, Iceland; karlsk@hi.is

**Keywords:** diphyllobothriosis, fish-borne zoonosis, short tandem repeats, polymorphic loci, microsatellite library screening

## Abstract

*Dibothriocephalus dendriticus* is one of the causative agents of the fish-borne zoonosis diphyllobothriosis. Polymorphic microsatellite markers were originally developed for future genetic studies using microsatellite library screening and next-generation sequencing (NGS). Out of 128 microsatellite candidates selected after NGS analysis, 126 yielded PCR products of the expected size. A declared repetitive motif was confirmed in 92 loci by Sanger sequencing. The level of polymorphism was tested by fragment analysis. Statistical tests for observed and expected heterozygosities and deviations from Hardy–Weinberg equilibrium revealed 14 polymorphic microsatellite loci suitable for studies on the finer genetic structure of global populations of *D. dendriticus*.

## 1. Introduction

Diphyllobothrioses are fish-borne parasitic zoonoses caused by tapeworms of the genera *Diphyllobothrium*, *Adenocephalus* and *Dibothriocephalus* (Cestoda: Diphyllobothriidea) [1]. *Dibothriocephalus dendriticus,* a previously underestimated causative agent of diphyllobothriosis, is the second most commonly reported *Dibothriocephalus* tapeworm from humans [2]. 

Its life cycle is complex, requiring three hosts for completion: (1) copepods act as the first intermediate hosts; (2) freshwater or anadromous fish are the second intermediate hosts; (3) and fish-eating birds and mammals, including humans, are the definitive hosts. The larval stages (plerocercoids) of *D. dendriticus,* which develop in the second intermediate fish hosts, have been found in more than 50 species from 12 families of freshwater fish [3]. However, most of the infections were recorded in salmonids in the northern hemisphere (e.g., rainbow trout *Oncorhynchus mykiss*, brown trout *Salmo trutta*, Arctic charr *Salvelinus alpinus*, Atlantic salmon *Salmo salar* and European whitefish *Coregonus lavaretus*) [2]. The definitive hosts of this euryxenous parasite are fish-eating birds (e.g., Laridae, Alcidae, Corvidae, Gaviidae, Podicipedidae, etc.) and mammals—especially canids (Arctic fox *Vulpes lagopus*, red fox *Vulpes vulpes*, domestic dog *Canis familiaris*), the brown bear (*Ursus arctos*) and the Eurasian otter (*Lutra lutra*) [2]. The definitive hosts get the infection per-orally by consuming plerocercoids that developed in the second intermediate fish hosts. 

Humans can be infected either by consuming raw or undercooked visceral organs (e.g., liver and ovaries) or fish fillets. Plerocercoids, normally encapsulated in viscera, may also occur in capsules attached to the ventral abdominal flaps or as free migrating worms in the musculature [4]. Since human infections have been considered as accidental, with mild or no symptoms, they may pass unnoticed and be underdiagnosed [2]. Human cases have been confirmed in Arctic North America (AK, USA; NU and BC, Canada), mostly in the Native Inuit population [5]. In Europe, human infections have been reported mainly in Russia, for example, in the Baikal and Siberia regions (for a review, see [2]). 

The original distribution of *D. dendriticus* is circumboreal, but it has also allegedly been found in second intermediate fish hosts in Argentina and Chile [2]. *D. dendriticus* has been detected in different species of fish and fish-eating mammals in Canada [6], Greenland [7], Iceland [8], the British Isles [9,10] and Russia [11]. The tapeworm has also been found in different fish throughout Scandinavia, in Finland [12], Norway [13,14] and Sweden [15]. 

The zoonotic potential, broad geographic distribution and wide spectrum of intermediate and definitive hosts make *D. dendriticus* an interesting parasitic model. Although numerous records on its life cycle, occurrence and ecology (see references above) have been published, studies on the origin, zoogeography, phylogeography and genetic interrelationships among populations are still missing. Until now, different subunits of nuclear ribosomal RNA genes and mitochondrial genes have been used as effective molecular tools for the taxonomy and phylogeny of the order Diphyllobothriidea [1]. Nevertheless, the application of these markers in population genetics has certain limitations due to their specific structure and mode of inheritance. Microsatellites, or short tandem repeats (STRs), are highly polymorphic multilocus markers distributed throughout the genome. They are popular tools in population genetics studies due to their characteristics, such as Mendelian inheritance, codominance, high allelic variation, locus specificity and wide genome coverage [16]. Recently, six microsatellite markers were developed for *Dibothriocephalus latus*, the type species of the genus and the most frequent causative agent of human diphyllobothriosis in the Holarctic region [17]. *D. latus* and *D. dendriticus* are phylogenetically closely related congeners [1] with similar life cycles involving two intermediate hosts and one definitive fish-eating host. We were interested in the structure and polymorphism of microsatellites in *D. dendriticus*, another medically important species of the genus. Hence, the aim of the work was to develop genetically informative STR markers for *D. dendriticus* as potential genetic tags for future studies.

## 2. Material and Methods

### 2.1. Sample Collection and Molecular Genotyping

The STR design was performed by a microsatellite library screening followed by several validation steps for which *D. dendriticus* plerocercoids from the following localities and hosts were applied: (i) Norway, Lake Takvatn (NO-TA), brown trout *Salmo trutta;* (ii) Norway, Lake Kalandsvatn (NO-KA), brown trout; (iii) Iceland, Lake Hafravatn (IS-HA), brown trout; (iv) Iceland, Lake Þingvallavatn (IS-PI), Arctic charr *Salvelinus alpinus.* Table 1 indicates the populations and number of individuals involved in the particular methodological steps. Molecular genotyping (PCR and sequencing) was performed on each individual larva for their exact taxonomic identification. A species-specific PCR amplifying partial mitochondrial cox1 was performed with the MulDen4 (5′-GTGTTTTTCATTTGATGATGACCAGTC-3′) and MulRevCom (5′-ATGATAAGGGAYAGGRGCYCA-3′) primers [18]. Each PCR product was sequenced using the BigDye Terminator v3.1 Cycle sequencing kit (Applied Biosystems, Foster City, CA, USA) and the Applied Biosystems 3130xl genetic analyser (Applied Biosystems). The sequences were analysed using the program Geneious, version 10.0.5 (Biomatters, Auckland, New Zealand). Only plerocercoids whose sequences displayed 100% identity with those of *D. dendriticus*, for example, Estonia, the United Kingdom and Russia (GenBank accession numbers GU997616-19 [18]) were included in the study.

### 2.2. Next-Generation Sequencing (NGS) Analysis by the GenoScreen

The microsatellite library screening was performed by the commercial NGS GenoSat^®^ service (GenoScreen, Lille, France). According to their recommendations, the genomic DNA was isolated from 11 *D. dendriticus* individuals (NO-TA population) using the QIAamp^®^ DNA Mini Kit (QIAGEN, Hilden, Germany), equimolarly mixed, cleaned and concentrated by the DNA Clean & Concentrator^TM^-5 (Zymo Research, Freiburg, Germany) to the final 2 μg of total DNA. The microsatellite library screening was performed through the Illumina MiSeq Nano 2 × 250 v2 of DNA libraries enriched for AG, AC, AAC, AAG, AGG, ACG, ACAT and ATCT repeat motifs. The obtained data were assembled using a Velvet assembler. The bioinformatics program QDD v3 [19] was used for the best remapping, sequence assembly and primers design. 

### 2.3. PCR Amplification and Sequencing 

The primers recommended by GenoScreen were applied to three *D. dendriticus* individuals (Table 1) in order to test the PCR amplification effectiveness of the designed markers. The total volume of the PCR mixture was 20 μL and contained 10–20 ng of genomic DNA, 10 pmol of each of the two primers, 0.2 mM of each of the deoxynucleotide triphosphate (Fermentas, Vilnius, Lithuania), 0.5 U of Taq DNA polymerase (Fermentas) with corresponding reaction buffer and 1.5 mM MgCl_2_. The amplification was performed in a Bio-Rad C1000™ thermal cycler programmed for 5 min at 94 °C as the initial step, followed by 30 cycles of 1 min at 94 °C, 1 min at 55 °C and 2 min at 72 °C. The final step was 5 min at 72 °C. The PCR products were visualized on 1.5% agarose gel. Only loci with the positive amplification of PCR products of expected sizes were considered for the further sequencing procedure.

In order to confirm the presence of a declared repetitive motif, PCR products were sequenced from both sides using the BigDye Terminator v3.1 Cycle sequencing kit (Applied Biosystems) and the ABI 3130xl Genetic Analyser (Applied Biosystems). The sequences obtained were visually checked for the presence of a declared repetitive motif using Geneious software version 10.0.5 (Biomatters).

### 2.4. Fragment Analysis and Population Genetic Statistics

For determination of STR allele polymorphism, fragment analysis with fluorescently labelled primers was performed under the following conditions: 1 μL of amplified PCR product was mixed with 8.5 μL of HiDi Formamide and 0.5 μL of GeneScan-LIZ500 Size Standard (both Applied Biosystems), and the mix was denatured for 5 min at 95 °C. Capillary electrophoresis was performed on an ABI 3130xl Genetic Analyser (Applied Biosystems), and the GeneMapper v3.7 software (Applied Biosystems) was used for genotyping. Statistical tests for the total number of alleles (Na), the effective number of alleles (Ne), observed (Ho) and expected heterozygosities (He), and significance values for Hardy–Weinberg equilibrium (HWE) were calculated for each locus and all populations using GenAlEx 6.5 [20].

## 3. Results

### 3.1. NGS Analysis

The following results were obtained after NGS analysis by the GenoScreen service: In total, 3,873,037 raw sequences were obtained and assembled in 51,983 contigs, 1639 sequences of which comprised repetitive motifs. Finally, 128 primer sets with the best resolution were designed and bioinformatically validated by GenoScreen for further PCR amplification of 128 STR loci. The designed primers amplified microsatellite regions spanning 90–300 bp; the most frequent repetitive motifs were dinucleotide repeats (71.9%), followed by tri- (25.8%) and tetranucleotide repeats (2.3%). 

### 3.2. Validation of Microsatellite Candidates

After STR loci selection and primer design, several validation steps were performed. The first one was PCR amplification, which was applied to test the amplification effectiveness of the designed primers. PCR products of expected size were detected in 126 out of 128 tested microsatellite candidates. The second validation step was Sanger sequencing aimed at the detection of the declared repetitive motifs. In total, 126 PCR products were sequenced and the repetitive motif was confirmed in 92 of them (Table 1). For determination of STR allele polymorphism, fragment analysis with fluorescently labelled primers was performed for 40 randomly selected loci on eight *D. dendriticus* individuals (NO-KA population; Table 1). Out of 40 tested STR loci, 23 were detected to be monomorphic or had to be excluded from further analyses due to a failure to amplify the PCR product (potential of homozygotes for null alleles). The remaining 17 microsatellite candidates were polymorphic and consequently subjected to a second round of fragment analysis. This was aimed at the final selection of loci and focused on an evaluation of their specificity and polymorphism on a broader sample set from geographically distant populations. In this validation step, six specimens from each *D. dendriticus* population (NO-TA and NO-KA from Norway; IS-HA and IS-PI from Iceland) were applied (see Table 1). Finally, 14 out of 17 loci consistently amplified PCR products in all 24 individuals. Table 2 summarizes the repeat motif and approximate size of the PCR products of the 14 selected STR loci (sequences provided in the Supplementary file S1) and the primers necessary for their amplification. 

Statistical tests for the total number of alleles (Na), the effective number of alleles (Ne), observed (Ho) and expected heterozygosities (He), and significance values for Hardy–Weinberg equilibrium (HWE) revealed the following results: In total, 1–8 and 1–11 alleles were detected in individual loci in the IS-HA and IS-PI populations from Iceland, respectively (percentage of polymorphic loci: 92.9%). In both populations, locus DD_38 was monomorphic, while locus DD_2 displayed the highest level of polymorphism (8 allelic variants in IS-HA and 11 in IS-PI) (Table 3). In the NO-KA population from Norway, all loci were polymorphic (2–5 alleles/locus), while in the NO-TA population loci DD_84 and DD_95 were monomorphic, and the rest of the loci contained 2–4 alleles/locus (percentage of polymorphic loci: 85.7%) (Table 3). 

The lowest values of observed heterozygosity were 0.17 (DD_23/IS-HA; DD_95/IS-PI; DD_84/NO-KA) and 0.2 (DD_25/IS-PI; DD_43/NO-TA). Equally frequent alleles, displayed by a 1.0 value of heterozygosity, were detected for locus DD_2 in IS-HA, IS-PI and NO-KA; for loci DD_23 and DD_84 in IS-PI; loci DD_38, DD_57 and DD_95 in NO-KA; and DD_47 in NO-TA. Tests for Hardy–Weinberg equilibrium revealed deviations from HWE in the Icelandic population IS-HA in four loci (DD_2, DD_23, DD_33 and DD_43) and in one locus (DD_38) in the Norwegian population NO-KA (Table 3). No significant differences were detected between the values of observed (Ho) and expected heterozygosities (He).

## 4. Discussion

The current results on *D. dendriticus* revealed a high number of sufficiently polymorphic loci, promising their future application in population genetics. On the contrary, rather different results were obtained after STR design in the congeneric tapeworm *D. latus.* While 23 out of 40 STR loci (57.5%) were monomorphic in the currently analysed *D. dendriticus*, as many as 72 out of 78 loci (92.3%) were detected as monomorphic in *D. latus* [17]. For *D. dendriticus*, 14 microsatellite candidates were finally validated for further application, but only six polymorphic STR loci were designed in *D. latus* [17]. The low level of polymorphism and heterozygosity in *D. latus* was related to self-fertilization as the most probable way of reproduction of this hermaphroditic tapeworm [17]. On the other hand, the presently detected higher level of genetic polymorphism in *D. dendriticus* indicates more frequent cross-fertilization or mixed mating between individuals. 

Although the primary reason for STR design for *D. dendriticus* and *D. latus* was the selection of sufficiently informative and highly polymorphic loci, the differences in the genetic variation and polymorphism indicate different ways of their reproduction as well [17; current study]. The probability of self-fertilization in *D. latus* and cross-fertilization in the studied *D. dendriticus* populations is also supported by their basic parasitological characteristics, such as the intensity of infection and prevalence. 

The principal second intermediate fish hosts of *D. latus* (the European perch *Perca fluviatilis* and Northern pike *Esox lucius*) are mainly infected by a single plerocercoid in the epaxial musculature [21]. In addition, a prevalence ranging between 2% and 37.5% has recently been detected in the Alpine lakes region, epidemiologically and ecologically one of the most important enzootic regions of *D. latus* in Europe [21]. Humans, who can be infected by consuming raw or undercooked fish fillets, represent the most important definitive host, and contribute significantly to the maintenance of the life cycle of *D. latus*. Other definitive hosts, mainly fish-eating Canidae (red fox *V. vulpes*, grey wolf *Canis lupus*, domestic dog *C. familiaris*) and Felidae (domestic cat *Felis catus*) acquire infection by the ingestion of fish infected with a *D. latus* plerocercoid. It is highly probable that the majority of definitive hosts are infected by the random consumption of a plerocercoid from one infected fish. The rarity of such infections suggests that mostly single plerocercoids develop into adult tapeworms in the definitive hosts. This is supported by the fact that *D. latus* has been found as a single worm in wolves [22] and humans [23]. 

Contrary to this, the encapsulated larval stages of *D. dendriticus* are present within the visceral organs or body cavity in fish in high numbers [2]. In our parasitological examinations of brown trout and Arctic charr from different lakes in Iceland, some fish harboured >200 plerocercoids; in addition, a 100% prevalence was detected in brown trout from the Hafravatn and Másvatn lakes [24]. Thus, fish-eating definitive hosts have a high probability of acquiring infections and being infected with many plerocercoids. Consequently, definitive hosts can harbour several adults reproducing by cross-fertilization. For example, a grizzly bear (British Columbia, Canada) was found to be infected with three *D. dendriticus* tapeworms [6], and two Arctic foxes from the coastal habitat in Iceland hosted four and eight *D. dendriticus* adults, respectively [8]. Hickey and Harris (1947) [25] studied *D. dendriticus* in different species of gulls (*Larus marinus*, *L. fuscus*, *L. argentatus*), in which the greatest number of adult specimens in a single bird was 11 and of immature tapeworms as many as 51.

The currently designed and validated microsatellite markers in *D. dendriticus* show a high level of polymorphism and are suitable for the detection of deeper and more detailed genetic structure of allopatric populations of this zoonotic tapeworm. The effectivity of their further application depends on a broad sample set of parasites coming from different geographic regions. Future analyses of STR loci polymorphism of world-wide populations could reveal if *D. dendriticus* is native to Northern Europe or if it is a result of transcontinental introduction from North America.

## Figures and Tables

**Table 1:** Details on different validation steps applied in development of microsatellite markers in *Dibothriocephalus dendriticus.*

Methodology	Purpose of the Method	No. T	No. S	Origin and Number of *Dd* Specimens Involved in the Analysis
Microsatellite library screening	Identification of candidate microsatellite loci	-	128	Norway; Lake Takvatn; brown trout *Salmo trutta*; 11 specimens
PCR amplification	Validation of amplification effectiveness of designed primers	128	126	Norway; Lake Takvatn; brown trout *S. trutta*; 3 specimens
Sanger sequencing	Confirmation of a presence of declared repetitive motifs	126	92	PCR products obtained after PCR amplification were sequenced
Fragment analysis	Primary testing of heterozygosity of candidate loci	40	17	Norway; Lake Kalandsvatn; brown trout *S. trutta*; 8 specimens
Statistical tests	Heterozygosity tests and calculation of HW equilibrium	17	14	Norway; Lake Takvatn; brown trout *S. trutta*; 6 specimens
				Norway; Lake Kalandsvatn; brown trout *S. trutta*; 6 specimens
				Iceland; Lake Hafravatn; brown trout *S. trutta*; 6 specimens
				Iceland; Lake Þingvallavatn; Arctic charr *Salvelinus alpinus*; 6 specimens

No. T, number of loci which were tested in the analysis; No. S, number of loci which were selected after analysis; Dd, *Dibothriocephalus dendriticus*; HW, Hardy–Weinberg.

**Table 2:** Characteristics of 14 microsatellite markers identified in *D. dendriticus*.

Locus	Forward Primer Sequence (5′-3′)	Reverse Primer Sequence (5′-3′)	Repeat Motif	PCR Product Size (bp) ^a^	Number of Alleles ^b^
Dd_2	CCGACAACAACGCTCTAATCC	TGCCATTCAGCAAGGTGGAA	(act)_n_	~210	19
Dd_17	ACGCTACTGCATAGATCGAGG	GCATAACGCGCCAGAAACAA	(ac)_n_	~240	5
Dd_23	CACACGCAGAAGTCTAGTTGAC	TGTTAGCTTACTTCCGTGGCT	(ac)_n_	~140	5
Dd_25	GTTATCCTACGTTGGGCTCCT	ATCTGGTTGGGAGAAACAACT	(ac)_n_	~90	3
Dd_33	TGTTTGCTCCAGTGCCTCG	CTAGCAGCATCAGCAGTGGA	(acgc)_n_	~270	6
Dd_38	ACTATCACGATGCGCTGACA	ATCCTTTGTTCCCTGAGCAG	(ag)_n_	~250	5
Dd_43	CAGTCTTTCCGGGTGAAGCT	GGTAGCTGCAGTACCGATCA	(aat)_n_	~210	8
Dd_47	ACTTCGGATTACTTCATTAACTCAGT	TGGTGAACGAAGTCAAACTATGC	(agg)_n_	~190	10
Dd_49	ACGTCTGACGACAACTTGGG	AAGACCCTGGCCAATACACG	(at)_n_	~190	6
Dd_57	AACATGCGAGTCCCAGGAAG	AGCAACGATCTACCGTAAAGCA	(aag)_n_	~120	8
Dd_78	GCTTTCGGCCATTTGTGGTC	GGGACAATAGGCAGGGTCTG	(ag)_n_	~270	4
Dd_84	AGAGGTAATTCATCGAGTTCTCTGA	TGACTGTGTACATCCGGTCG	(agg)_n_	~240	6
Dd_95	CGTTCACGCTCCAATGATCC	AGAGCTTGCTGATGATGGCT	(ag)_n_	~190	3
Dd_114	ACTTCAGGTAATCTCCGTGTCC	CTAGCGCCAATGGGTAGCTT	(aaat)_n_	~130	5

^a^ The exact size of PCR products depends on the number of repetitive motifs detected in the particular *D. dendriticus* individual; ^b^ the total number of different allelic variants detected in the particular microsatellite locus in four geographically distant populations tested in the current study.

**Table 3:** Statistical data on 14 microsatellite markers validated in studied *D. dendriticus* populations.

*D. dendriticus* Population	Locus	Na	Ne	Ho	He	uHe	DF	Signif.
Iceland	DD_2	8	6.00	1.00	0.83	0.91	28	*
Lake Hafravatn	DD_17	3	1.67	0.50	0.40	0.44	3	ns
(IS-HA)	DD_23	3	2.67	0.17	0.63	0.68	3	*
	DD_25	2	1.60	0.50	0.38	0.41	1	ns
	DD_33	3	2.88	0.50	0.65	0.71	3	*
	DD_38	1	1.00	0.00	0.00	0.00	x	x
	DD_43	3	2.88	0.50	0.65	0.71	3	*
	DD_47	5	3.79	0.67	0.74	0.80	10	ns
	DD_49	4	3.27	0.67	0.69	0.76	6	ns
	DD_57	3	2.32	0.33	0.57	0.62	3	ns
	DD_78	4	2.48	0.50	0.60	0.65	6	ns
	DD_84	4	3.43	0.83	0.71	0.77	6	ns
	DD_95	2	1.47	0.40	0.32	0.36	1	ns
	DD_114	3	2.88	0.83	0.65	0.71	3	ns
Iceland	DD_2	11	10.29	1.00	0.90	0.98	55	ns
Lake Þingvallavatn	DD_17	3	2.67	0.67	0.63	0.68	3	ns
(IS-PI)	DD_23	3	2.32	1.00	0.57	0.62	3	ns
	DD_25	3	2.17	0.20	0.54	0.60	3	ns
	DD_33	3	1.67	0.50	0.40	0.44	3	ns
	DD_38	1	1.00	0.00	0.00	0.00	x	x
	DD_43	6	3.79	0.67	0.74	0.80	15	ns
	DD_47	6	4.17	0.80	0.76	0.84	15	ns
	DD_49	4	1.71	0.33	0.42	0.45	6	ns
	DD_57	7	5.54	0.67	0.82	0.89	21	ns
	DD_78	4	2.40	0.50	0.58	0.64	6	ns
	DD_84	5	4.00	1.00	0.75	0.82	10	ns
	DD_95	2	1.60	0.17	0.38	0.41	1	ns
	DD_114	3	2.67	0.83	0.63	0.68	3	ns
Norway	DD_2	5	4.50	1.00	0.78	0.85	10	ns
Lake Kalandsvatn	DD_17	4	3.79	0.83	0.74	0.80	6	ns
(NO-KA)	DD_23	3	2.18	0.67	0.54	0.59	3	ns
	DD_25	2	1.80	0.67	0.44	0.48	1	ns
	DD_33	3	1.95	0.67	0.49	0.53	3	ns
	DD_38	4	4.00	1.00	0.75	0.82	6	**
	DD_43	4	3.43	0.67	0.71	0.77	6	ns
	DD_47	5	3.79	0.83	0.74	0.80	10	ns
	DD_49	3	2.67	0.83	0.63	0.68	3	ns
	DD_57	4	3.27	1.00	0.69	0.76	6	ns
	DD_78	3	2.32	0.83	0.57	0.62	3	ns
	DD_84	2	1.18	0.17	0.15	0.17	1	ns
	DD_95	3	2.67	1.00	0.63	0.68	3	ns
	DD_114	3	2.32	0.83	0.57	0.62	3	ns
Norway	DD_2	4	3.27	0.83	0.69	0.76	6	ns
Lake Takvatn	DD_17	3	1.85	0.60	0.46	0.51	3	ns
(NO-TA)	DD_23	3	3.00	0.83	0.67	0.73	3	ns
	DD_25	2	1.80	0.33	0.44	0.48	1	ns
	DD_33	2	1.38	0.33	0.28	0.30	1	ns
	DD_38	3	2.18	0.83	0.54	0.59	3	ns
	DD_43	3	1.85	0.20	0.46	0.51	3	ns
	DD_47	4	3.13	1.00	0.68	0.74	6	ns
	DD_49	3	2.57	0.67	0.61	0.67	3	ns
	DD_57	3	2.00	0.67	0.50	0.55	3	ns
	DD_78	3	1.67	0.33	0.40	0.44	3	ns
	DD_84	1	1.00	0.00	0.00	0.00	x	x
	DD_95	1	1.00	0.00	0.00	0.00	x	x
	DD_114	2	1.60	0.50	0.38	0.41	1	ns

Na, number of different alleles detected for particular locus and *D. dendriticus* population; Ne, number of effective alleles; Ho, observed heterozygosity; He, expected heterozygosity; uHe, unbiased expected heterozygosity; DF, degrees of freedom; Signif., significance values for Hardy–Weinberg equilibrium test; ns, not significant; *, *p* < 0.05; **, *p* < 0.01; x, monomorphic loci for which tests were not performed.

## Supplementary Materials

The following is available online at https://www.mdpi.com/2073-4425/11/7/782/s1, File S1: Sequences of 14 microsatellite markers identified in *Dibothriocephalus dendriticus* provided after the microsatellite library screening by the GenoScreen.
