# Supplementary material for: Development of 14 Microsatellite Markers for Zoonotic Tapeworm Dibothriocephalus dendriticus (Cestoda: Diphyllobothriidea)
Source: Genes (Basel). 2020 Jul 12;11(7):782. doi: 10.3390/genes11070782 (PMC7397143; doi:10.3390/genes11070782)
Supplement: Supplementary file 1 [file genes-11-00782-s001.pdf]

Supplementary file Sequences of 14 microsatellite markers identified in *Dibothriocephalus dendriticus* provided after the microsatellite library screening by the GenoScreen. Declared repeat motifs are highlighted by red/bold.

Locus Dd_2; Repeat motif **ACT**
TCATTATTCATGTGAAACTATGCATTGATTCACCATTAACGAATCCGTGTACGGACATATACTGAAACTTCAAGAGAGAGAGCTCCAAGACCAT
AATCAGTATTTTCAAAGGAACTTACGTTATTTCCCCACCCTAGCTTCTAAAGGAGTTTCTTCATATAAAAGAAACCCAAAACCCCGACAACAACG
CTCTAATCCACGAAAACCGCGAAAGTCTAGTTACTTCGAGCTTCGAATTCGTCGGTGTATTGCGTCAAGCCCTCTAG**ACTACTACTACTACTACT**
ACTACTACTACTACTACTACTACTACTACTACTACTACCTTTGGTGCCAATGATGTCCCTGAAACACGAGACAAAACTACTCAGCAGTTCCACCT
TGCTGAATGGCACTTGTTTTCTGGCTCCCGGATTATTCCCAGTTCTGAAAGGTTAACAATACCACCAGGACTAACTGATAACTATGTTAGGTATC
TTCAATTTGTTTGCGGTGCGGGAATATCGCCTACCGGAAAAGGGCATTACGGAACACGAATAACGGGCGACAGGTGATAACGCGACGTCCCA
GTAACTGTCTAGGTCCGCCTAACTGT

Locus Dd_17; Repeat motif **AC**
CCGTGAGATTTAACGTATCATGTTACGTAAAGTAAAAAATTCTGAATTCACATCTCAGTTGAATCACCTGGGCTTTCTCTCTTTACTCGCATAAC
GCGCCAGAAACAAACCGTAGTTGGGCAGACTAGCTGTAGTGGAGTTGTCTTACGGGCTGGCATGACATAAATCATTTGTCGAAAGCGTAACA
TAGGTTAAAAGCC**ACACACACACACACACAC**TATAGCCCTAGTCGTTGATAAGGACAAGTTCTATTAAGAGCCGATAAATATTGCAGTAGTTG
GTTCTGCGTGTCTCATGTGTCTGATTGGGCCTCGATCTATGCAGTAGCGTGTAGACGCAGTTCCGAAAAATGGAGGGAAGACTAGACATATCC
TGGTCTGATGTCCGCGCACTGAGTCTCCAGCTGTGCTGCAATGGA

Locus Dd_23; Repeat motif **AC**
ATCTGCTTGCTAGAATGAACGTGCTCTTTCCGGTTTGGATGGAAGGACTTCGGTTTGAAGATAAAATGTTTTACCCAGCATGTCTCATCAGTGA
ATCCCTCACATCTATACCCTAACGAGTGAGATTGGTGTAAAAAGAGACAATGATAGCACGACTTTCGCTACTTTCAGACTGGACAGACTAGGT
GTTATGGTTACCTCTGTGTCGTATTCTGCCCATAGACGTAGAGTCGGGGGACGATTTGCAGACCAGACACTCGTATTTGCAAACACCAACCGG
AAGCTCTCTCTCTCACACGCAGAAGTCTAGTTGACGTAGACTGTTTACCTTTAGCAGACGGCCCGCGCATATGCGC**ACACACACACACACACAC**
GATAGCCAGTGACTATTGTTCACTACAGTAGAAGTTTACCAAAGCCACGGAAGTAAGCTAACAGGGTAAAAAGTTCAAAGACAACCGAACTAT
CATCTGCCTGCTCCTCACGCCAG

Locus Dd_25; Repeat motif **AC**
GGATGCAGAGACTGCCAGATCGGCTCCTCTCCCCCCCTTCGACCTACCAGCTGCAGCTGTTCATTTACCCAGCCCACGCCCCCTCCCCCCCGGA
GGGGGGAAGCTATAGGTGCACGGATAAGGGGGTGTCGGCCGGCAAAATGACAGCGGCGATGAGGGGGGGGCGGAACTGTCAAGTACGAC
TTATGATATCTCTGCGAACCTGCCTATCGATAGTATCCTCTAAACGCGCACTGAGGACTGTTAGGAATGCGGTTGCAGCCAAGAGGGTACAAC
GCGGCGCAACCGCAAAATTGTCCCATTTAAGAGTGCAGATTTATGTTATCCTACGTTGGGCTCCTAGGGGTGTGGGAGACTTCAA**ACACACAC**
ACACACACACGCATGCGTTATAGTTGTTTCTCCCAACCAGATATCTCAT

Locus Dd_33; Repeat motif **ACGC**
GCGAAGAGATATCAAAGACGACGCGCGACGGACTAGCAGCATCAGCAGTGGAGGGCAAGCGCGCGCGGGTAAGATCAGAGTAGTTGCAGT
AAGAGGCAGAGGACGATGAGGCACGCCGGCGATTTAGCGCACAATAGAGTTTACGCACGGGC**ACGCACGCACGCACGCACGCACGCACGC**
ACGCGTTGCAGAATATGAACAGCAGCGCAGCGCAGCGCAGCTACGCTTAGGCGCGGTTGACGCCCACGAGAGTGAGAGAGAAATAAAACAG
GTTGTTTTGCAGTCGCCCGAGGCACTGGAGCAAACAGGGGGAGTAAATGAGCGGCAGAAGTGGTTCAGGTATGGCCCGTCATGTGCGGGAT
AGTTTTGCAGGTGTATATCGGAGTTTGCCCGCCCGGCTTTTAGAGCTCTCGTATCACTCAGATCTACCCG

Locus Dd_38; Repeat motif **AG**
GGCACATTCATCGTTATGAAAAAGTTCAAGACGCATTGAAACTATCACGATGCGCTGACAAGATAAGTAGCTCCTGTCAGG**AGAGAGAGAGA**
GAGACCGTTAGACGGCGGTAGATCCTTCTGAACATGGTCTTGACGACTTAAAGGGAAAGCGTTTTCACAAAGAAGAAGAGATCAAGATACAA
TGACTGGCGGCACTTGACACCATTAGCTGCGTTTATGGTCCTCTCTATCGACCGTGCCTGTCGACAAAGAGGCAATATGCAAACCTGCTCAGG
GAACAAAGGATAAGTGTGTGTGTAATACATAGCCGTATTACACACGAACATAATCCTACCTCGATCAACTGTGTTCTTTAGGGTCAACATTCGT
TGCCGGGTTAATAAGTTAGCAAGATAGTTTACTGGCTTTATCGCAGAGGGTTATGTGAGTGAAGCCCTAGCAACTGCGATCTTCAGACCATGG
TTCCTTGGGAGATAAAAGAGAAGTCTTGGGAAGGCCAGAGTTTTAGGAATAGTGTATTCCTCACAGAAGTATAGGACAATAAATGTAAAGAG
TTTTGACTCGCGGATGACTGCAGTCCGACTGCGAGGGTGTCAATTCCGGAGTGACGAGAGCGT

Locus Dd_43; Repeat motif **AAT**
AGGCATATGCTCAGTCTTTCCGGGTGAAGCTTTTGGAACAGAATATAAGATTTATAGACCCTCAGAGGAGAGGAGATGACAATTTCTCCCACT
TGAGGTGTACAGTGTATAAAG**AATAATAATAATAATAATAATAAT**AAAAGTTCTCCTGCTCTCCTTTTGCTGACACGTGCAATTCAGCTAAAAC
TATCTATATAGAACGTTTTGATCGGTACTGCAGCTACCGGGGAATCTTGTAAAATTTAACCAAAATTTGAAATGCGATTTCAAGTAGGAAGAAT
TTATTGCATGGAGTTGTGTCTAAGGATTATCACGTTACCTTATCGCAGGATATTTCTGTCACGCCAAGATGCGGGCTTTTGAATAAAACGGGGT
TGTTAGAGTCTGATTTGAAGGTAAGGACGGCATTAGGCTTTGTACAACA

Locus Dd_47; Repeat motif **AGG**

AGTCGACTTTCGAAAAAGGAAGATGGACAAGTAACATACAGTATAACCCAGATGGAAAATTTTCCCATTTGGAGTAGACTTACCCTGGGAATA
AACCGATTGGCAGATGAAATGAAAGCAGGTTATGAAATAGCCAAAAAAACCTTCAGTTATATACGCTCATAGATAAATGGATATGTTCAACCA
ATTAAAAAACGAAAAGATTTTTCTAGCATCTTAGGTAAAGCTACATATGGTGAACGAAGTCAAACTATGCTGATTTCCACGATGTCATGGACAA
CAAATATGCAATAGAACGATTCCGTTGTAGAAGTTAAGGAGGAGGAGGAGAAGGAGGAGA**AGGAGGAGGAGGAGGAGGAGGAGG**AGAA
ACCCAAGTCAACCACTGACCTAGTATATCAACTGAGTTAATGAAGTAATCCGAAGT

Locus Dd_49; Repeat motif **AT**
GGTGCGACCGATACAGACTGGCGCAAACTGCAGCCGCTACGAAGCGCTCGCACCAAACAAATATTTATTCTTTCGGTACTTTTGAAATTTCAAT
TGTTCTCTACACACTTACTCACGTCTGACGACAACTTGGGCATCCGGCATTAAAACATTTACCCAATAAAGTTATCTTGTTCCCCGAAGAACTTC
TCTTATTTACA**ATATATATATATATATAT**AAGCATGTACGAACTTGCCTGCTGTGCTGCAGTTGCGGAGACGGCCACTATTGGGGCGTCAGCGAA
ACGTGTATTGGCCAGGGTCTTGGATATCCGACTTTTCATCTGAAAACAGACTTGCAGTAAGTGTGAAGGCATGAGACAAACGCACCTTGGCAA
TCTTCTCCTCTCGTGTGCTCGGCTCAAGTAGGTCCACCTTG

Locus Dd_57; Repeat motif **AAG**
TTGTAACTTGGACGTCAGGGAAGGACAACTGATTTTTGACATTTCTTCCATCGTAAACAGAATTTCGGAAAAAAATTGGCTTCATTAATGCCTT
GAAAGCTCTTGCCCTACCTTGACATCTAAGTAACAAAACTGGCAGACGGGAAAATAAAAGCAACGATCTACCGTAAAGCAACTAACACCAGAC
ACATTCTTCACGTAG**AAGAAGAAGAAGAAGAAGAAG**ACCCGATCGCTGGGACAATACGTTTATTCACTTCCTGGGACTCGCATGTTCGCTTG
TATTGACATTCTTCTCTTCCAAAGCAACCACTGTGTTTGTCACAAACGCAGTTGTGTCCAAACCCTCCTCCGACGTGTTCAGGCACACTGTAGCG
ACGACAGTGGGAAGCGAGAGGAGGTGAAGTACTTTTATACCCTTTTCAAA

Locus Dd_78; Repeat motif **AG**
TTGTGGGGCGATTGAGTGCGTTAATAAGGTGACTGCATGCTGAATGGAGGGACAATAGGCAGGGTCTGTTGAAGGGGACAGGACGAGAGTT
CGCCCATGGGACCGGTCCATGCATGTGCCCATTGTAAGCCAGTCTTGTCCACGTCATAGGCGGATTTGAGGGAGAGAAAGAGGGAAAGAGG
GTGGGAGAGGGAGGTAC**AGAGAGAGAGAGAG**ATGCGTTTTACTTTGAAAGGATCATTCTAAAGAATTCATCCATTCACTGTGGACAGAATA
GGGTAAGTAAAACAACTAACACCGGAAGACCACAAATGGCCGAAAGCCACAACTGTCAACATTAAAAACACTCGCCGACAAGTGAGTCACAC
AATTTACGGCAGATTTCACATGCTTCAAAACGCTGCTAACGTGGTGTGC

Locus Dd_84; Repeat motif **AGG**
TACCCTATTCCGTGAAGAGGAGGTTGCAACAAATGCATGCGAACTGTTCCCCGGCTGTGGCAACTATGTGCTCTGCGCCCTGTTCGCTGAACC
GCGCTCGAAGCTGAGGCAACTAGAGGACGACCCATTAGATGGGTCAGTGACCTTGGAGGACTTTTTCAAGCTGAGTTGTGAAATTGACTGTG
TACATCCGGTCGATGGCCATCAATCAGTTGTTGTGACGGAGGGAGGAGAGAACGAGCAGACGGAGGAGGACTTCGAGGGAAATGAATTCAT
CTCTAGTGCGCCGCCCTCGAGTTGCGCCAGTGGACAAATCAACAGAGAAGAGCGTGCAGAAGGGG**AGGAGGAGGAGGAGGAGG**ACAGTG
ATGCAGACGCGAGTGAAGGTAGAAGAGCATCAGAGAACTCGATGAATTACCTCTAC

Locus Dd_95; Repeat motif **AG**
TTTTCGTAGGTGATTGGTGATCGTTCATTCGCAAATATCAAACTTTCTGATCTAAACCTGCAAGCGTTGGGCTTCTACTCAGCAGGTCATGGGTT
CGAGTCTTGAGTTATGCACTACATGGACTTGAGTAGAGCTTGCTGATGATGGCTAGAAATGGCTATTCCAGATGCTGTTAAACTCAAACGTCG
GGAAGTGAGAAGG**AGAGAGAGAGAG**ATACACTTTATTTTGAAGGTGGCATCCAAAATTTTAAGCAAAACGCGGCTTATATGGAGGTTCGAC
GGACTGCGTTCTTGCAGGGGATCATTGGAGCGTGAACGGATTCCCTGACGGGGTGGTCCTTTCAGATACTGTTGAGACGTTTAAACGCTAACT
GAATTCTCATTTGCTGAGAAACTGTGATAGATATAATGGACA

Locus Dd_114; Repeat motif **AAAT**
GCCCCTTTGTTATTATCATCTTGTCCTAATTTTATCTTTAAAGAGGAAAATTTTATTACAAGGATGTTTTAGTTACTGAACACTGCATTCAAACTG
CCAAAGGCTTTTTAGGGTTAATTCCCAAGGGAACGATATGCATGAAGACAGCTTCGAGCTAGCGTACCCAATTTTTAGTTCACGAATTTGCTAG
CGCCAATGGGTAGCTTTAGTGTCCACCCGAAAGGACCTAGTTCTCAACAT**AAATAAATAAATAAATAAATAAAT**AAACATTTTGACCGAACCT
ACATCACTACTCTAGGACACGGAGATTACCTGAAGTCAATCCCTTGATTTAAATAAAAGAGGTGATAGAACAAATGCGTTGTCACCAAATTACC
AAAAGATTCCCGACCCTAAAAAACGGCATCTGCAGAAAAAAGAGCACAATA
